# The Effect of Transitioning between Feeding Methods on the Gut Microbiota Dynamics of Yaks on the Qinghai–Tibet Plateau

**DOI:** 10.3390/ani10091641

**Published:** 2020-09-11

**Authors:** Xiao-Ling Zhang, Tian-Wei Xu, Xun-Gang Wang, Yuan-Yue Geng, Hong-Jin Liu, Lin-Yong Hu, Na Zhao, Sheng-Ping Kang, Wan-Min Zhang, Shi-Xiao Xu

**Affiliations:** 1 Northwest Institute of Plateau Biology, Chinese Academy of Sciences, Xining 810008, China; 18362985573@163.com (X.-L.Z.); xutianwei@nwipb.cas.cn (T.-W.X.); wangxgucas@163.com (X.-G.W.); gengyuanyue19@mails.ucas.ac.cn (Y.-Y.G.); liuhj@nwipb.cas.cn (H.-J.L.); xiangchou812@163.com (L.-Y.H.); zhao88na@163.com (N.Z.); kangsp@nwipb.cas.cn (S.-P.K.); 2 University of Chinese Academy of Sciences, Beijing 100049, China; 3 Haibei Demonstration Zone of Plateau Modern Ecological Animal Husbandry Science and Technology, Haibei 812200, China; z88wanm@163.com

**Keywords:** gut microbiota, adaptation time, dynamic variation, yak, Qinghai–Tibet Plateau

## Abstract

**Simple Summary:**

This study explores the gut microbiota alterations that occur when transferring yaks from winter grassland to feedlot feeding, and to determine the adaptation period. Our results demonstrated that such transferring could influence the gut micro-ecology, and was stabilized within 16 days. This study will improve the understanding of the processes behind gut microbiota adaptation to an abrupt change in feeding methods, and will provide a reasonable adaptation period for yak management, which could be applied to nutritional research and minimize detrimental effects in the animals.

**Abstract:**

Here we aimed to explore the change in yak gut microbiota after transferring yaks from grazing grassland to a feedlot, and determine their diet adaptation period. Five yaks were transferred from winter pasture to an indoor feedlot. Fecal samples were obtained from grazing (G) and feedlot feeding yaks at day 1 (D1), day 4 (D4), day 7 (D7), day 11 (D11), and day 16 (D16). The dynamic variation of the bacterial community was analyzed using 16S rRNA gene sequencing. The results showed that the yak gut microbial community structure underwent significant changes after diet transition. At the phylum and genus levels, most bacteria changed within D1–D11; however, no significant changes were observed from D11–D16. Furthermore, we used random forest to determine the key bacteria (at class level) disturbing gut micro-ecology. The relative abundance of the top four classes (*Erysipelotrichia*, *Gammaproteobacteria*, *Saccharimonadia*, and *Coriobacteriia*) was highest on D1–D4, and then decreased and plateaued over time. Our results demonstrated that an abrupt adjustment to a diet with high nutrition could influence the gut micro-ecology, which was stabilized within 16 days, thus providing insights into diet adaptation in the yak gut.

## 1. Introduction

Yaks (*Bos grunniens*, Linnaeus 1766) that live on the Qinghai–Tibet Plateau (QTP) at an altitude of more than 3000 m have good adaptability to live in high-altitude and low-oxygen environment. Yaks are the critical resource species on the QTP, promoting the local economy. As such, yaks are an ideal model for the study of nutrition in livestock on the QTP. In recent years, due to the promotion of sustainable and healthy development of ecological animal husbandry, yak feeding management has gradually shifted from grazing all year round to grazing in the warm season and feedlot feeding in the cold season [1]. However, many pastoralists’ traditional nomadic ideas are deeply rooted, and animal husbandry on the QTP still faces many problems, such as grassland degeneration and the seasonal imbalance of naturally nutritious pasture [2,3,4]. Thus, reasonable nutrition management is extremely important for improving the farmed yak performance. In order to determine a reasonable and healthy feeding management scheme, a nutritional trial needs to be conducted. This trial should account for adaptation time, as a reasonable adaptation period can minimize the potential impact carried over from a previous management [5]. The main challenge is that the eating behavior, physiology, and metabolism of the ruminant, especially the gastrointestinal microbes, must adapt to the new feeding scheme in just a few days or weeks [6]. An abrupt change in the nutritional content may adversely influence the animals, resulting in metabolic disturbances. For yak gastrointestinal microbiota research, researchers have focused on effect of different nutritional levels [7], but overlooked an abrupt diet change from one feeding management to another. Therefore, it is essential to explore how the yak gut microbiota adapt to an abrupt diet change, and explore the threshold of this adaption.

Some studies on the adaptation period of ruminants have been reported, but the researchers only paid attention to the ruminant digestion, fermentation, and rumen microbiota, and were less focused on the dynamics of intestinal microbiota [5,8,9]. However, the intestinal microbiota plays a crucial role in animal health and food safety, as they regulate the host’s metabolism through various functions, and the diversity in the microbial community structure is affected by changes in diet [10,11]. Therefore, understanding the effect of an abrupt change in diet on the gut microbiota is essential.

Given the importance of yaks on the QTP, this study focused on the changes and adaptability in the yak’s gut microbiota when adapting from a low nutrition (grazing) diet to a higher nutritional (feedlot feeding) diet. We used 16S rRNA sequencing to understand the dynamics of gut microbiota during an adaptation of period of 16 days, and employed the random forest model to search for key bacterial changes. This investigation will improve understanding of the processes behind gut microbiota adaptation to an abrupt change in feeding methods, and will provide a reasonable adaptation period for yak management, which could be applied to nutritional research and minimize detrimental effects in the animals.

## 2. Materials and Methods

### 2.1. Animal Selection and Management

This study was conducted in January 2019 at the Haibei Demonstration Zone of Plateau Modern Ecological Animal Husbandry Science and Technology, in Qinghai Province, China. All procedures involving animal care were consistent with the guidelines provided by the Institution of Animal Care and the Ethics Committee of the Northwest Institute of Plateau Biology, Chinese Academy of Sciences (NWIPB20160302).

A total of five 2-year-old healthy female yaks (body weight: 107.83 ± 4.26 kg) were selected from the grazing grassland. Ear tags were put on each yak, and the yaks were randomly allocated into individual pens. The trial process was as follows: collection of the required samples at pasture (G), then transfer of the yaks into the feedlot for a 16-day feeding period (D). The diet was formulated according to the Nation Research Council (NRC) [12]; the nutrient composition is shown in Table 1. During the trial, yaks were allowed free access to water and fed twice daily, at 8:00 and 17:00, with feeding at 1% of their body weight in the first few days, which was then gradually increased.

### 2.2. Sampling and Measurement

The contents of crude proteins (CP), neutral detergent fiber (NDF), acid detergent fiber (ADF), Ca, and P in each sample were measured in laboratory, and the metabolizable energy (ME) was calculated. A total of 100 g mixed feed was collected and dried in a forced-air oven at 60 °C for 48 h, then ground through a 1 mm sieve before being analyzed. The dry matter (DM) and N contents were determined according to the Association of Official Analytical Chemists (AOAC) [13]. The NDF and ADF contents were analyzed according to Van Soest et al. [14].

The main objective in this study was to determine the composition and stability of the gut microbiota over a short duration (16 days). In order to determine this, fresh fecal matter is required. The sampling procedure for fresh fecal matter was as follows: the G group’s samples were obtained in the morning before feeding, while the D group samples were obtained every morning before 8:00 on feedlot feeding day 1 (D1), day 4 (D4), day7 (D7), day11 (D11), and day 16 (D16). Disposable sterile gloves were worn for sample collection to avoid pollution. A total of 30 samples were collected and placed in 5 mL frozen tubes to avoid cross-contamination between them. The samples were immediately frozen in liquid nitrogen and stored at −80 °C.

### 2.3. DNA Extraction, 16S rRNA Gene Amplification, and Sequencing

Genomic DNA was extracted from the fecal samples using a MN NucleoSpin 96 Soil Kit (Macherey-Nagel, Düren, Germany) according to the manufacturer’s protocol. The quality and concentration of the extracted DNA were measured using a NanoDrop spectrophotometer (ND-1000, NanoDrop Technologies, Wilmington, DE, USA). The V3–V4 region of the 16S rDNA gene was PCR-amplified (98 °C for 2 min, followed by 30 cycles of 98 °C for 30 s, 50 °C for 30 s, 72 °C for 1 min, and 72 °C for 5 min) using the primers 338F (5′-ACTCCTACGGGAGGCAGCA-3′) and 806R (5′-GGACTACHVGGVTWTCTAAT-3′). Barcodes were added to the ends of the primers. The PCR products were mixed with equivalent volumes of 2 loading buffer were subjected to 1.8% agarose gel electrophoresis for detection. Samples with a bright main band of approximately 450 bp were chosen and mixed in equidensity rations. Then, the mixture of PCR products was purified using a GeneJET Gel Extraction Kit (Thermo Fisher Scientific, Waltham, MA, USA). Sequencing libraries were validated using an Agilent 2100 Bioanalyzer (Agilent Technologies, Palo Alto, CA, USA) and verified with a Qubit 2.0 Fluorometer (Thermo Fisher). Finally, pair-end sequencing was conducted using an Illumina HiSeq 2500 platform (Illumina, Inc., San Diego, CA, USA). All raw sequences obtained were submitted to the National Center for Biotechnology Information (NCBI) Sequence Read Archive (accession number: SUB7228971).

### 2.4. Bioinformatics Analysis

The overlapping regions between the paired-end reads were merged using FLASH v1.2.7, and row reads were quality-filtered under specific filtering conditions to obtain high-quality clean tags on the Trimmomatic v0.33. The chimera sequences were detected by comparing tags with the reference database (Ribosomal Database Program) using the UCHIME v4.2 algorithm, and then removed. The effective sequences were then used in the final analysis.

Sequences were grouped into operation taxonomic units (OTUs) using the clustering program UCLUST [15] against the SILVA v1.2.11 bacterial database [16], pre-clustered at 97% sequence identity. Taxonomic information for each representative sequence was annotated using the Ribosomal Database Program (RDP) classifier (v16).

Beta diversity was calculated using Bray–Curtis and principal coordinates analysis (PCoA). The comparison of microbiota was performed by an Adonis() function in the vegan package. Correlations between different groups were accessed by Spearman’s correlation analysis using a cor() function in the corrpolt package. Significant differences in each groups’ intestinal flora were analyzed by MetaStats, and a line chart was prepared using Origin 2018.

We regressed the relative abundances of bacterial taxa at the class level against time using the default parameters for the R implementation of the algorithm (R package “randomForest”, ntree = 1000, using default entry of *p*/3, where *p* is the number of taxa of the class). Lists of taxa ranked by random forest in order of feature importance were determined over 100 iterations. Then the number of marker taxa were identified using 10-fold cross-validation implemented with the rfcv() function in the R package “randomForest”, and graphed using the ggplot2() package in R.

## 3. Results

### 3.1. 16S rRNA Gene Sequencing of the Gut Bacteria

**Figure 1:**
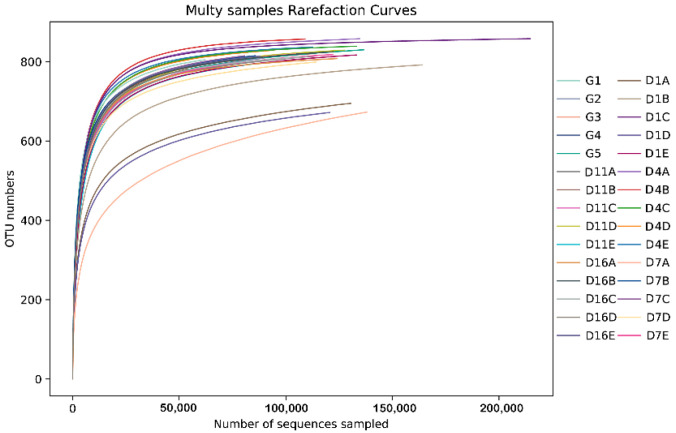
Rarefaction curves obtained by plotting the number of operational taxon units (OTUs) by number of reads for all fecal samples. The *x*-axis shows the number of valid sequences per sample and the *y*-axis shows the number of observed species (in OTUs).

A total of 5,156,882 effective tags were obtained from the 30 fecal samples, as well as 171,896 ± 44,715 (range: 75,119–314,485) analyzed sequences (mean length = 411.5 bp) from each sample. A total of 25,054 OTUs were obtained based on a sequence-similarity level of 97%, with 808.19 ± 47.89 (range: 672–888) as the mean number of OTUs per sample. The rarefaction curves for the OTUs detected in this study showed that the quality of observed species increased as the sequencing depth increased. The ends of the rarefaction curves tapered off with increasing numbers of sequences per sample, as is commonly observed with sequencing data (Figure 1). Post-filtering sequencing data is displayed in Supplementary Table S1. Classification of the OTUs resulted in the detected bacteria being classified into 10 phyla, 16 classes, 20 orders, 49 families, and 142 genera.

### 3.2. Beta Diversity and Correlation Analysis of the Gut Bacteria

**Figure 2:**
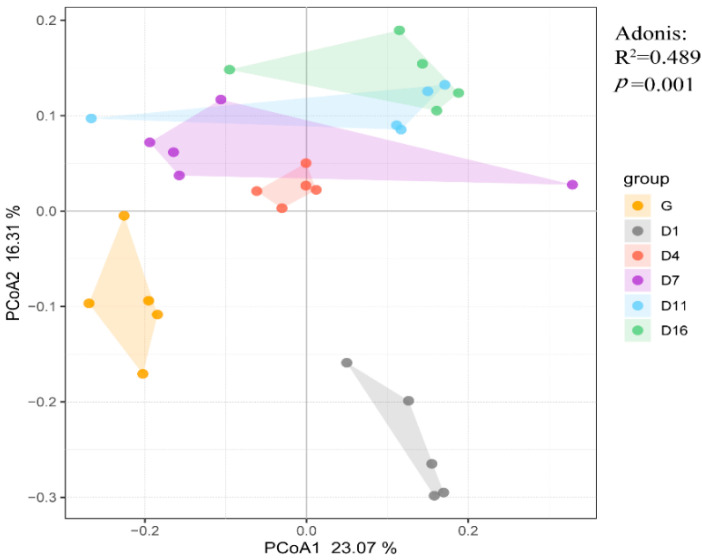
Principal coordinated analysis (PCoA) using Bray–Curtis dissimilarity, based on genus-level OTUs from yak fecal samples collected from the G (grazing) group and D1, D4, D7, D11, and D16 (feedlot feeding days 1, 4, 7, 11, and 16, respectively).

Principal coordinated analysis (PCoA) analysis of overall diversity based on Bray–Curtis was performed to compare the six groups (Figure 2). We can see a shift in the gut microbial population structure by transition of the diet, and such a shift was also observed in D1 and D4–D16, but the distance between the groups gradually decreased from D4. Most samples clustered together according to their particular group, suggesting that each group hosts its own distinct bacterial community. Adonis (PERMANOVA) showed this alteration to be statistically significant (*R*^2^ = 0.489, *p* = 0.001).

The Spearman correlation test is shown in Table 2. The correlation between G and D1 was relatively high compared to others; the other D groups gradually decreased over time when compared to the G group. During the feedlot feeding period, the correlation between each group fluctuated, but a trend of overall increase was observed. The Spearman correlation coefficient increased from 0.57 (D1 and D4) to 0.79 (D11 and D16) between two adjacent groups.

### 3.3. Taxonomic Analysis of the Gut Bacteria

**Figure 3:**
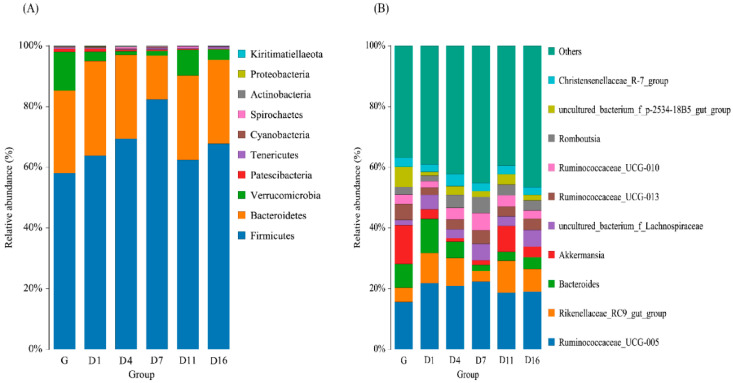
Phylum (**A**) and genus (**B**) (relative abundance) composition for fecal samples collected from G (grazing) group and D1, D4, D7, D11, and D16 (feedlot feeding days 1, 4, 7, 11, and 16, respectively).

**Figure 4:**
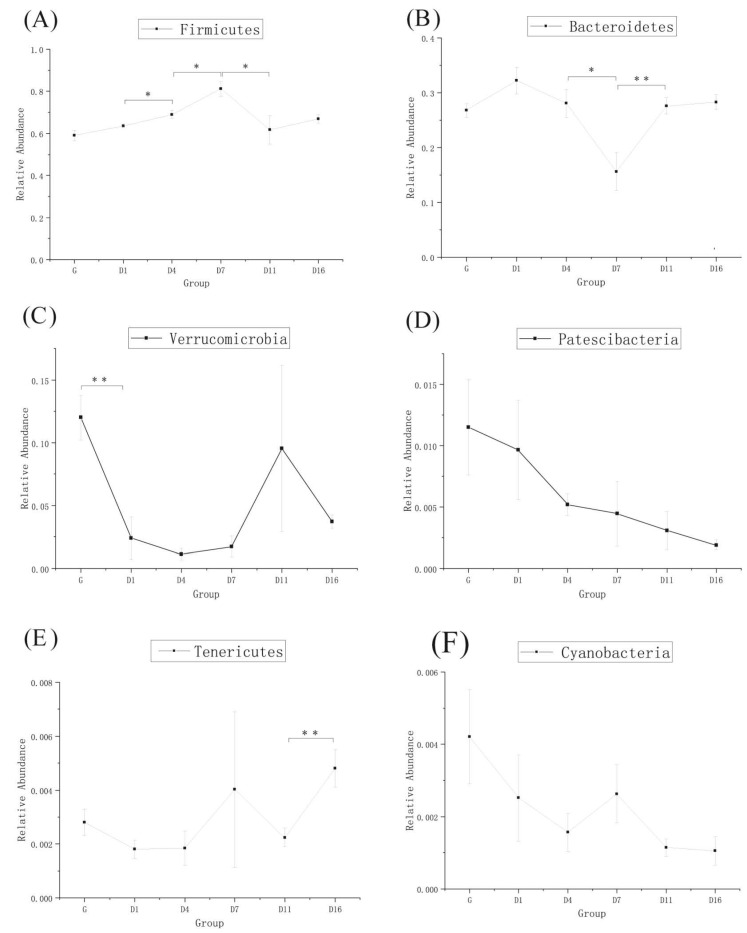
MetaStats analysis of top phylum bacterial of yak, (**A**–**F**) are the top six phylum, and * means *p*-value < 0.05, ** means *p*-value < 0.01. (G: grazing; D1, D4, D7, D11, and D16: feedlot feeding days 1, 4, 7, 11, and 16, respectively).

**Figure 5:**
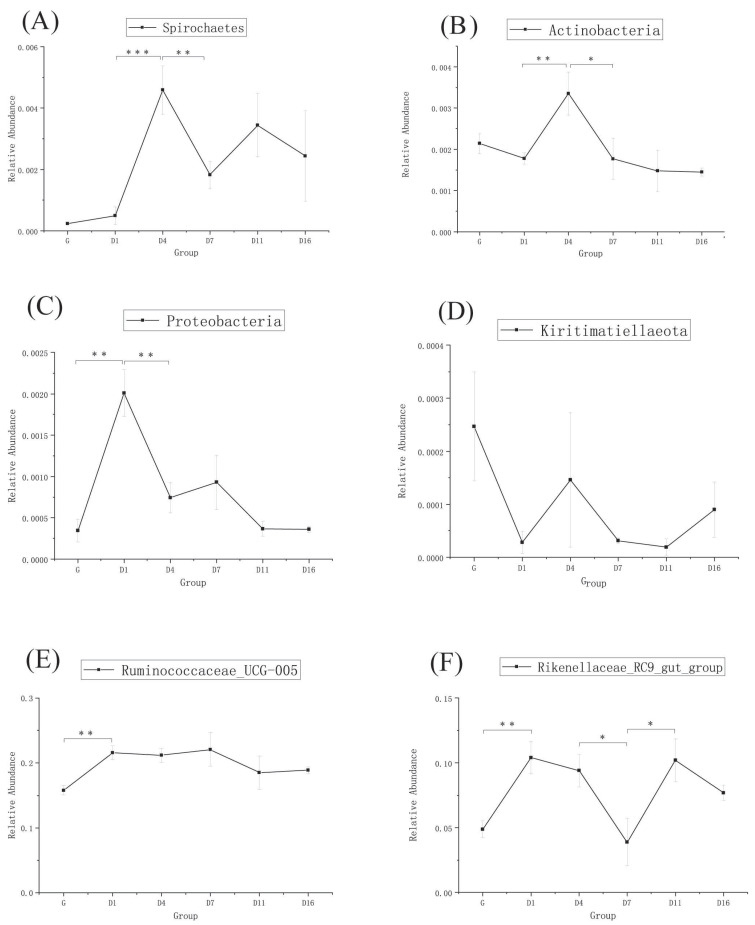
MetaStats analysis of top phylum and genus bacterial of yak: (**A**–**D**) phylum and (**E**,**F**) genera, and * means *p*-value < 0.05, ** means *p*-value < 0.01, *** means *p*-value < 0.001. (G: grazing; D1, D4, D7, D11, and D16: feedlot feeding days 1, 4, 7, 11, and 16, respectively).

The taxonomic analysis at the phylum level is shown in Figure 3A. *Firmicutes*, *Bacteroidetes*, and *Verrucomicrobia* were the dominant phyla, and their abundance changed most obviously in each group. From Figure 4A–F and Figure 5A–D, the relative abundance of *Firmicutes* significantly increased in D4 and D7, then decreased in D11 (*p* < 0.05). *Bacteroidetes* were lowest in D7, but significantly increased in D11 (*p* < 0.01). *Verrucomicrobia* was significantly decreased in D1, then had a sudden increase in D11, but became reduced again in D16. *Patescibacteria* and *Cyanobacteria* were higher in the grazing group, whereas these two phyla showed no significant change during the whole period. *Proteobacteria* was highest in D1, but was significantly decreased in D4 (*p* < 0.01). *Actinobacteria* was significantly increased in D4 (*p* < 0.01), but decreased in D7 (*p* < 0.05). Overall, there was no significant changes observed in most of the detected bacteria between D11 and D16 except *Tenericutes*.

The taxonomic analysis at the genus level is shown in Figure 3B. *Ruminococcaceae_UCG-005* and *Rikenellaceae_RC9_gut_group* were the most predominant genera in each group. *Ruminococcaceae_UCG-005* significantly increased in D1, but no significant changes were observed over the following few days. *Rikenellaceae_RC9_gut_group* significantly increased in D1, then significantly decreased in D7, but significantly increased again in D11. Both of these two genera had no significant changes in the D11 and D16 groups (Figure 5E,F).

### 3.4. Random Forest Regression Analysis

**Figure 6:**
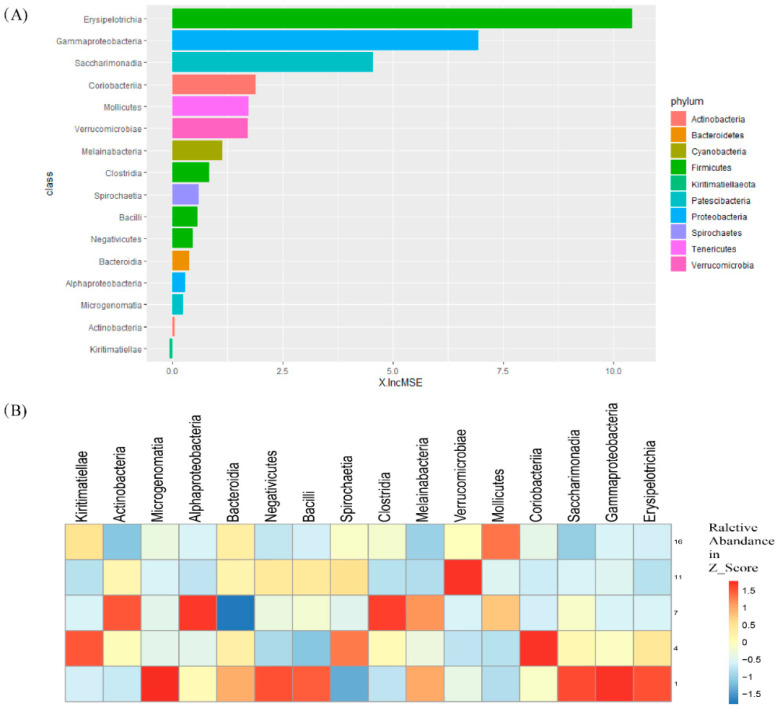
The key bacteria of feedlot feeding periods (D1, D4, D7, D11, D16) in yaks. (**A**) The key bacteria in class were identified by applying random forest regression. (**B**) Heatmap showing the changes in relative abundances of bacteria ordered by importance in feedlot feeding period.

In order to determine the key bacteria involved in breaking the gut micro-ecology stability, we used a random forest machine learning algorithm. To establish a model that correlated gut microbiota with time in the D groups, the same diet was applied in each group, so that only the effect of time would be observed. The model explained 72.75% of the gut microbiota variance related to the adaptation of time. To reveal the importance of bacterial classes as key bacteria taxa in the whole adaptation period, we performed a 10-fold cross-validation. The results showed that the error rate was lowest when the feature was equal to 16. All 16 classes are shown in Figure 6A according to time-discriminatory importance. *Erysipelotrichia* (*Firmicutes*), *Gammaproteobacteria* (*proteobacteria*), *Saccharimonadia* (*Patescibacteria*), *Coriobacteriia* (*Actinobacteria*), and *Mollicutes* (*Tenericutes*) were the top five biomarkers based on class level. Furthermore, the majority of bacteria showed higher relative abundance from D1–D7, and the relative abundance decreased after that (Figure 6B). There was little change observed from D11 to D16 in most of the classes, especially *Erysipelotrichia*, *Gammaproteobacteria*, *Saccharimonadia*, and *Coriobacteriia*, which had a higher increase in mean squared error (%, %IncMSE). Some bacteria still exhibited changes in D11–D16, like *Mollicutes* and *verrucomicrobiae*; however, they had few changes from D1–D7, which is contrary to that observed of other bacteria.

## 4. Discussion

A change in feeding method means change in diet, and diet is one of the main factors affecting the gut microbial community. Weimer et al. [17] found that dietary changes in cannulated cattle caused changes in the rumen bacterial community that would rebuild within 10 days. In our study, the gut microbial community structure of yaks was significantly affected by the abrupt transition of their diet. During the study, the cluster between G and D1 was separate from each other as well as the other groups, but from D4 these distances in each group gradually minimized. It is well established the gut microbiota play an important role in animal digestion, and diet is a vital factor that shapes microbial communities [18,19]. Therefore, when yaks move from the pasture to the barn, the original stability of the gut bacteria is broken, as a result of the interaction between nutrients and microbiota. Microbiota have adapted the ability to absorb energy from specific nutritional content with an added competitive edge; as such, they rely on the nutritional stability of the gut, which affects their relative abundance [20]. Furthermore, correlation analysis showed the gut microbiota correlation between grazing and feedlot feeding decreased over time, while the correlation between each group of the feedlot feeding period gradually increased over time. This indicates that the gut microbiota gradually stabilized over time, which is in accordance with the PCoA.

Anderson et al. [21] found that diet affected the rate and duration of the animal’s adjustment period, and rumen bacteria adapted to high concentrate diet faster than traditional adaptation programs. For cattle, abruptly transitioning from a forage-based diet to a cereal-based diet can precipitate a host of metabolic disorders, and this may have long-term consequences [22,23]. In nutritional trials, to prevent the ruminants from acute acid poison or other such diseases, a reasonable transition period is required, as this is a critical factor for optimizing the diet before beginning the trial [24]. On the other hand, a reasonable adaptation time is another important factor in the growth performance for the animals. In the present study, we found that regardless of the fluctuations in the phyla bacteria during the earlier days after diet transition, no significant changes were observed in D11~D16. This was especially demonstrated in the *Firmicutes* and *Bacteroidetes* with the highest relative abundance, and similar results showed in the top two genus bacteria. This suggests that most of the gut microbiota adapted within 16 days after diet transition. This result is consistent with the previous convention that 2 weeks of adaptation can minimize the potential impact of diet changes on animals and remove carry-over from the previous treatment. Barducci et al. [25] reported that the rumen papillae were still adapting at 9 days, but after 14 days of adaptation, the rumen epithelium was totally adapted. Machado et al. [5] reported an adaptation period of two weeks for the bacterial community to stabilize under a new diet. In another trial, the adaptation of Nellore at 14 days and 21 days was compared, and it was found that although the rumen wall absorptive surface area was larger at 21 days than at 14 days, Nellore yearling bulls could become adapted by 14 days [26]. These findings support the two-week adaptive period required when transitioning between feeding methods.

Callaway et al. [27] and de Menezes et al. [28] found that *Firmicutes* and *Bacteroidetes* are the most abundant phyla in the gut microbiota of ruminants. They play a critical role in the microbial ecology of the mammalian gut, and are thought to be involved in the decomposition of fibrous and non-fibrous diets [29,30,31]. In our study, *Firmicutes*, *Bacteroidetes*, and *Verrucomicrobia* were the dominant phyla, which is in agreement with the previous studies. However, these dominant bacteria phyla displayed different tendencies subsequent to feedlot feeding. The proportion of *Firmicutes* significantly increased by D4 and D7, then decreased by D11. While the *Bacteroidetes* decreased from D4–D7, then significantly increased by D11. The previous study showed the relative abundance of *Firmicutes* increased in high nutrition diets [32], on account of the high energy providing for the microbiota and accelerating their breeding. Meanwhile, *Bacteroidetes* showed a decrease, suggesting that they cannot compete in the first few days after diet transition [33]. *Tenericutes* exists in many mammal guts; it includes both beneficial and pathogenic bacterial taxa [34]. In our study, only this phyla significantly changed during D11–D16; it appeared to be a lag response. Although the relative abundance of *Proteobacteria* was not high enough, it also plays an important role in gut microbiota. A previous study mentioned that *Proteobacteria* is regarded as a core microbiota in digesting soluble carbohydrates, but this was in the rumen microbiota rather than the gut microbiota [24], so the active of taxa in *Proteobacteria* may differ. Shin et al. [35] found *Proteobacteria* to be an indicators of gut microbiota disorder; in our study, the *Proteobacteria* increased in D1 and decreased by D4, and *Actinobacteria* increased in D4. In previous studies, certain *Actinobacteria* produce antibiotics that kill pathogenic bacteria [36,37]. This may connote that the sudden transition of diet had adverse effects on the animals, and they self-regulated this unstable state. However, both these phyla, *Proteobacteria* and *Actinobacteria*, contain beneficial and pathogenic bacterial taxa, so this still needs further investigation. Less is known about *Verrucomicrobia*, another common phylum, but it appears to be a common component of the ruminant gut microbiota [38,39]. *Ruminococcaceae_UCG-005* and *Rikenellaceae_RC9_gut_group*, common inhabitants of the feces of many ruminants, were the most predominant genera. *Ruminococcaceae_UCG-005* belongs to the *Ruminococcaceae* family, and is involved in fiber digestion [40]. In our study, this genus was increased in D1 and maintained a stable relative abundance from D1–D16, which indicates that a new diet with relatively high nutrition (CP or energy) may promote its reproduction in order to decompose more nutrients. *Rikenellaceae_RC9_gut_group* is from the *Rikenellaceae* family; this family is indicative of good gastrointestinal health, and can facilitate the breakdown of proteins and carbohydrates in the diet [24,41]. In our study, *Rikenellaceae_RC9_gut_group* was significantly increased in D1, because the new diet has a higher content of proteins and carbohydrates than herbage content, but it significantly decreased by D7, and then subsequently increased to higher levels by D11. The reasons behind this require further study. These varying changes in bacteria may be due to the different responsive capacities of each bacteria to the dietary transition, but most of them were able to adapt to the new diet within 16 days. Through concrete analysis of the gut microbiota, the variations observed here provide a more detailed insight into the gut microbiota adaptability after diet changed.

The ruminant gastrointestinal tract begins during birth and progresses until a climactic community in a dynamic stable state is established, until environment, diet, or other factors change it [42]. Despite the above information on the dynamics of microbiota in the phylum and genus, we cannot know their importance yet. A random forest model in machine learning can identify the key bacteria that disrupt the gut micro-ecology stability and identify which microbiota cause intestinal disorders after diet transition. Through this analysis, *Erysipelotrichia*, *Gammaproteobacteria*, *Saccharimonadia*, *Coriobacteriia*, and *Mollicutes* were identified as the top five microbiotas. Their relative abundance, except for *Mollicutes*, was highest in D1 or D4, then gradually decreased before plateauing during D11 to D16. This indicates that the dominant microbiota become stable within 16 days. A previous study suggested that *Erysipelotrichia* and *Gammaproteobacteria* are closely related to an inflammatory response [43,44], while *Mollicutes* have been reported to have potential pathogenicity [34]. As previously discussed, the change in *Proteobacteria* and *Actinobacteria* may be related to gut microbiota self-regulation. On the genus level, *Gammaproteobacteria* belongs to *Proteobacteria*, and *Coriobacteriia* belongs to *Actinobacteria*. *Coriobacteriia* is effective against some diseases [37], and this information further proves the supposition. In summary, the main cause of gut micro-ecology disorder is inflammation caused by microbiota. The relative abundance of the bacteria is low in a stable environment, but increases when the gut micro-ecology becomes unstable, and this disadvantages the host. Nevertheless, in our study, the bacteria with high relative abundance were still beneficial bacteria (like *Ruminococcaceae_UCG-005* and *Rikenellaceae_RC9_gut_group*), and most of the pathogenic bacteria decreased by D11–D16. Thus, a yak’s gut microbiota can adapt to the transition between diets with large nutritional differences within about 16 days. This gives them the ability to adapt to a wide range of plant sources, in order to obtain enough nutrition and energy in the harsh condition of the QTP. However, this does not fully explain the yak’s stabilization, and a comprehensive analysis that combines other aspects of the yak metabolism should be carried out, as the animal may have a different adaptation duration under different diets or environment.

## 5. Conclusions

This study tracked the dynamic changes and relative stability of the yak gut microbes when transitioning between feeding methods (from grazing to feedlot feeding). Our results showed that the microbial community structure of the yak gut changes significantly during the transition, but will stabilize with time. On the phylum and genus levels, bacteria with relatively high relative abundance changed significantly in first 7 days, and most bacteria tended to stabilize by 11–16 days. Most of the class bacteria that make a large contribution to the gut microbiota, screened using random forest, also became stable by 11–16 days. These results provide an understanding of the diversity, phylum, and genus level taxa, as well as the class bacteria with higher contributions to adaptability of the microbiota in dietary transition. This helps to determine the adaptation time of the bacterial community, and thus provides guidance for yak feeding methods and diet optimization.

## Figures and Tables

**Table 1:** Ingredients and nutrient composition of the diet.

Ingredients (%)		Nutrient Composition (%)	
Oat hay	40.0	DM ^†^	83.50
Corn	24.6	CP	13.87
Wheat bran	15.6	ME MJ/kg ^‡^	11.95
Rapeseed meal	0.6	NDF	33.14
Corn meal	7.8	ADF	18.08
Soybean meal	9.0	Ca	0.73
Salt	0.6	P	0.59
Premix ^§^	0.6		
CaHPO_4_	0.6		
CaCO_3_	0.6		
Total	100.0		

DM: dry matter; CP: crude protein; NDF: neutral detergent fiber; ADF: acid detergent fiber. ^†^ DM is determined based on an air-dry basis. ^‡^ ME (metabolizable energy) = total digestible nutrients × 0.04409 × 0.82, according to the National Research Council [12]. ^§^ Premix (provided per kilogram of complete diet): vitamin A = 200,000 IU; vitamin D3 = 15,000 IU; vitamin E = 1250 IU; Cu = 375 mg; Fe = 15,000 mg; Zn = 750 mg; Mn = 1000 mg; Se = 7.5 mg.

**Table 2:** Spearman correlation test reveals similarities between groups: G (grazing) group and D1, D4, D7, D11, and D16 (feedlot feeding days 1, 4, 7, 11, and 16, respectively).

	G	D1	D4	D7	D11	D16
G	1.00					
D1	0.64	1.00				
D4	0.57	0.57	1.00			
D7	0.53	0.44	0.60	1.00		
D11	0.43	0.51	0.59	0.61	1.00	
D16	0.41	0.56	0.56	0.53	0.79	1.00

## Supplementary Materials

The following are available online at https://www.mdpi.com/2076-2615/10/9/1641/s1, Table S1: Statistical table of post-filtering sequencing data.
