# Supplementary material for: The Effect of Transitioning between Feeding Methods on the Gut Microbiota Dynamics of Yaks on the Qinghai–Tibet Plateau
Source: Animals (Basel). 2020 Sep 11;10(9):1641. doi: 10.3390/ani10091641 (PMC7552143; doi:10.3390/ani10091641)
Supplement: Supplementary file 1 [file animals-10-01641-s001.zip › animals-908478-supplementary.docx]

| **Sample ID** | **PE Reads** | **Clean Tags** | **Effective Tags** | **AvgLen(bp)** | **GC(%)** | **Effective(%)** |
| --- | --- | --- | --- | --- | --- | --- |
| G1 | 99571 | 90509 | 88311 | 412 | 52.64 | 88.69 |
| G2 | 145771 | 133088 | 127958 | 412 | 52.72 | 87.78 |
| G3 | 186521 | 170964 | 166098 | 412 | 52.75 | 89.05 |
| G4 | 196472 | 178147 | 171289 | 411 | 52.72 | 87.18 |
| G5 | 231722 | 211659 | 206323 | 413 | 52.49 | 89.04 |
| D1A | 225291 | 207114 | 171702 | 413 | 52.47 | 87.57 |
| D1B | 261781 | 236022 | 165514 | 413 | 52.43 | 86.9 |
| D1C | 361090 | 328548 | 160974 | 411 | 52.84 | 87.09 |
| D1D | 199378 | 181183 | 189427 | 413 | 52.32 | 87.12 |
| D1E | 241802 | 219876 | 129629 | 413 | 52.43 | 87.09 |
| D4A | 235648 | 215538 | 190914 | 410 | 53.02 | 87.16 |
| D4B | 198487 | 178820 | 199208 | 412 | 52.81 | 85.95 |
| D4C | 237930 | 215732 | 177646 | 412 | 52.84 | 86.47 |
| D4D | 163000 | 148042 | 75119 | 412 | 52.81 | 87.66 |
| D4E | 207841 | 185417 | 143568 | 412 | 52.91 | 85.57 |
| D7A | 237348 | 214812 | 197296 | 408 | 53.28 | 83.35 |
| D7B | 141084 | 127312 | 227495 | 411 | 52.73 | 88.17 |
| D7C | 149681 | 136788 | 314485 | 410 | 52.91 | 86.89 |
| D7D | 157880 | 145287 | 173695 | 408 | 53.2 | 89.97 |
| D7E | 196509 | 179182 | 210575 | 409 | 53.16 | 87.95 |
| D11A | 196909 | 179492 | 205390 | 411 | 53.02 | 87.2 |
| D11B | 189606 | 173270 | 170605 | 411 | 52.84 | 87.29 |
| D11C | 188047 | 168745 | 205743 | 412 | 52.84 | 85.6 |
| D11D | 219414 | 196737 | 142883 | 413 | 52.53 | 86.33 |
| D11E | 147978 | 133081 | 177857 | 413 | 52.6 | 87.6 |
| D16A | 216700 | 201397 | 197838 | 411 | 52.97 | 88.1 |
| D16B | 227742 | 212389 | 124396 | 411 | 52.85 | 87.47 |
| D16C | 203995 | 188527 | 130065 | 412 | 52.84 | 87.08 |
| D16D | 84864 | 78123 | 142046 | 413 | 52.51 | 88.52 |

Table S1. Statistical table of post-filtering sequencing data.

Sample: Name of sequencing sample (G: Grazing; D1, D4, D7, D11, D16: Feedlot feeding days 1, 4, 7, 11, and 16); Paired-End (PE) reads: Number of original PE reads; clean Tags: Number of valid sequences after removal of chimeras; AveLen (bp): Average length of valid sequences; GC (%): GC content of valid data; Effective (%): The percentage of valid sequences after chimera removal over the original number of PE reads.
